# The color of aerosol particles

**DOI:** 10.1038/s41598-023-28823-6

**Published:** 2023-01-28

**Authors:** Ramesh Giri, Matthew J. Berg

**Affiliations:** 1 Department of Physics, Kansas State University, 1228 North Martin Luther King Jr. Dr., Manhattan, KS 66502-2601 USA; 2 Corning Incorporated, 60 O’Connor Rd., Fairport, NY 14450 USA

**Keywords:** Optical sensors, Environmental impact

## Abstract

Digital in-line holography (DIH) is an established method to image small particles in a manner where image reconstruction is performed computationally post-measurement. This ability renders it ideal for aerosol characterization, where particle collection or confinement is often difficult, if not impossible. Conventional DIH provides a gray-scale image akin to a particle’s silhouette, and while it gives the particle size and shape, there is little information about the particle material. Based on the recognition that the spectral reflectance of a surface is partly determined by the material, we demonstrate a method to image free-flowing particles with DIH in color with the eventual aim to differentiate materials based on the observed color. Holograms formed by the weak backscattered light from individual particles illuminated by red, green, and blue lasers are recorded by a color sensor. Images are reconstructed from the holograms and then layered to form a color image, the color content of which is quantified by chromaticity analysis to establish a representative signature. A variety of mineral dust aerosols are studied where the different signatures suggest the possibility to differentiate particle material. The ability of the method to resolve the inhomogeneous composition within a single particle in some cases is shown as well.

## Introduction

Aerosols are ubiquitous in the natural and built-environment^1,2^. Understanding their physical and chemical properties is important for atmospheric science, manufacturing, and health among other applications. Combustion particles, wind-blown mineral dust, and water droplets in the atmosphere absorb and scatter sunlight leading to affects on the climate^3–5^. Mineral dust and biological particles can nucleate cloud droplets or ice^6–8^ and combustion particles negatively impact human health^9^. Useful materials such as carbon black, pigments, powders, and coatings are synthesized from aerosols^10^. Pathogens can spread as aerosols, causing disease in agricultural crops, livestock, and humans^11,12^. The analysis of exhaled aerosols allows the detection of some diseases^13^ and certain therapeutic drugs are efficiently delivered as aerosols^14,15^. Whatever the context, there remains a need for improved methods to characterize aerosols.

The characterization of aerosols can mean the measurement of particle size, shape, material composition and phase, density, and the concentration of particles. Particle size and shape are frequently among the most important and the diversity of aerosols in this sense can be vast. Atmospheric aerosols are a good example where particle size can range from 15 nm soot to mineral dust and cloud ice larger than 100 $\upmu$m^1,4,16^. Particle shape and composition can vary from the simple, uniform spherical particles of unperturbed water droplets to the inhomogeneous and highly complex shapes of mineral dust. Electron microscopy is perhaps the only method capable of observing this range of particle size and shape. Yet, to do so requires particle collection and sample preparation, thus preventing real-time analysis. The collection may be impossible or impractical as is often the case in the atmosphere, e.g., consider cloud ice and particles residing at high altitudes. As such, there is often the preference, if not the requirement, that aerosol characterization be conducted in contact-free, or in situ, manner.

No method exists that can characterize aerosols in situ across the full range of particle size and shape encountered in many contexts. Sizing can be achieved by electrostatic, mechanical, and optical means in a contact-free way, although shape determination is often ambiguous^1^. Light scattering methods are particularly capable wherein sizing is possible, with varying degrees of accuracy, for particles nanometers^17^ to hundreds of micrometers in size^18,19^. One way to do so is to illuminate a particle by a laser beam and measure the intensity of scattered light as a function of angle, resulting in a scattering pattern. It is even possible in some cases to *infer* the shape of particles with scattering. If a particle is known to be a uniform sphere, e.g., a water droplet, then it is also possible to measure the particle’s refractive index^20–22^. And, the recent integration of machine learning methods suggests the possibility of classifying the shapes of nonspherical particles using features in scattering patterns^23,24^.

However, conventional light-scattering methods suffer a fundamental limitation known as the inverse problem. Measured quantities, such as the scattering pattern, cannot be *uniquely* associated with a particle property of interest, like size or shape, without supplemental information or invoking assumptions^25^. This point has motivated work on a related method, digital holography, which is largely free of the inverse problem, at least with regard to particle size and shape. In its simplest embodiment, so-called digital in-line holography (DIH) involves illuminating a particle by a collimated laser beam expanded to approximately one centimeter in diameter. A small portion of the beam scatters from the particle, which then interferes with the unscattered portion across an image sensor, such as a CCD or CMOS array. The measured interference pattern is the hologram, from which an image of the particle can be computationally rendered and takes the form of the particle’s silhouette along the illumination direction. Provided it is well resolved, the image can reveal the particle’s size and (projected) shape far more accurately than conventional scattering methods.

Digital holography appears in a range of applications and many excellent reviews are available^26–29^. Perhaps the most common application is to stationary particles on transparent substrates, where the method is often called digital holographic microscopy (DHM)^28^. Particles in motion can also be examined with DHM provided they are entrained in a fluid or confined in an optical or electrodynamic trap. Because DHM involves the use of lenses between the particles and sensor, it is necessary to control the motion such that well-resolved holograms are captured. As such, DHM is difficult to apply to aerosols in an in situ way since the motion of *free-flowing* particles is difficult to control; lens-free DIH is then the simplest approach to take. A number of examples illustrate the usefulness of DIH for aerosol characterization in the laboratory^30–38^, industrial settings^39^, the outdoor environment^40,41^, from research aircraft^42–44^, and from a small unmanned areal vehicle^45^.

While digital holography can yield a particle’s size and shape, it conveys almost no information about the material. This is because the image is essentially a silhouette due to the opaque nature of many solid particles. The desire to know even the most basic information about particle material motivates an attempt to realize such sensitivity within a DIH approach. The concept we pursue involves forming a color image of a particle from holograms recorded with red, green, and blue laser light. Recognizing that other wavelengths, even those beyond the visible spectrum, could be used, we call the method Multi-Wavelength Digital Holography (MWDH). Here, the holograms are formed from backscattered (reflected) light rather than forward scattered (transmitted) light as in conventional DIH or DHM. While color digital holography is not new and its application to aerosols may appear elementary, the inherent motion of the particles significantly complicates the optical approach required and diminishes the image resolution compared to conventional methods. As will be seen, the new method permits the differentiation of particles by composition provided they exhibit significantly different colors. We also find some evidence for the resolution of inhomogeneities in the composition of a single aerosol particle.

## Imaging principle

Perhaps the most straightforward way to realize color holography is demonstrated by Garcia-Sucerquia^46^. Stationary particles are illuminated by red, green, and blue lasers in a transmission-mode DHM configuration and a color image is rendered by layering the images reconstructed from the holograms in each color. The method performs well because the particle is a $10\,\upmu$m section of a fixed biological specimen that is largely transparent to the light. However, for many solid aerosol particles, e.g., mineral dust, the material is typically opaque. Examination with a conventional white-light microscope shows that the particle color is far more distinct with epi-illumination compared to trans-illumination. Thus, we devise a method to render color images by forming the holograms with the light backscattered by the particle in the three primary colors. Because the spectral reflectance of a surface is partly dependent on the material, the approach has the potential to differentiate particles of different material, at least in a coarse manner.

**Figure 1:**
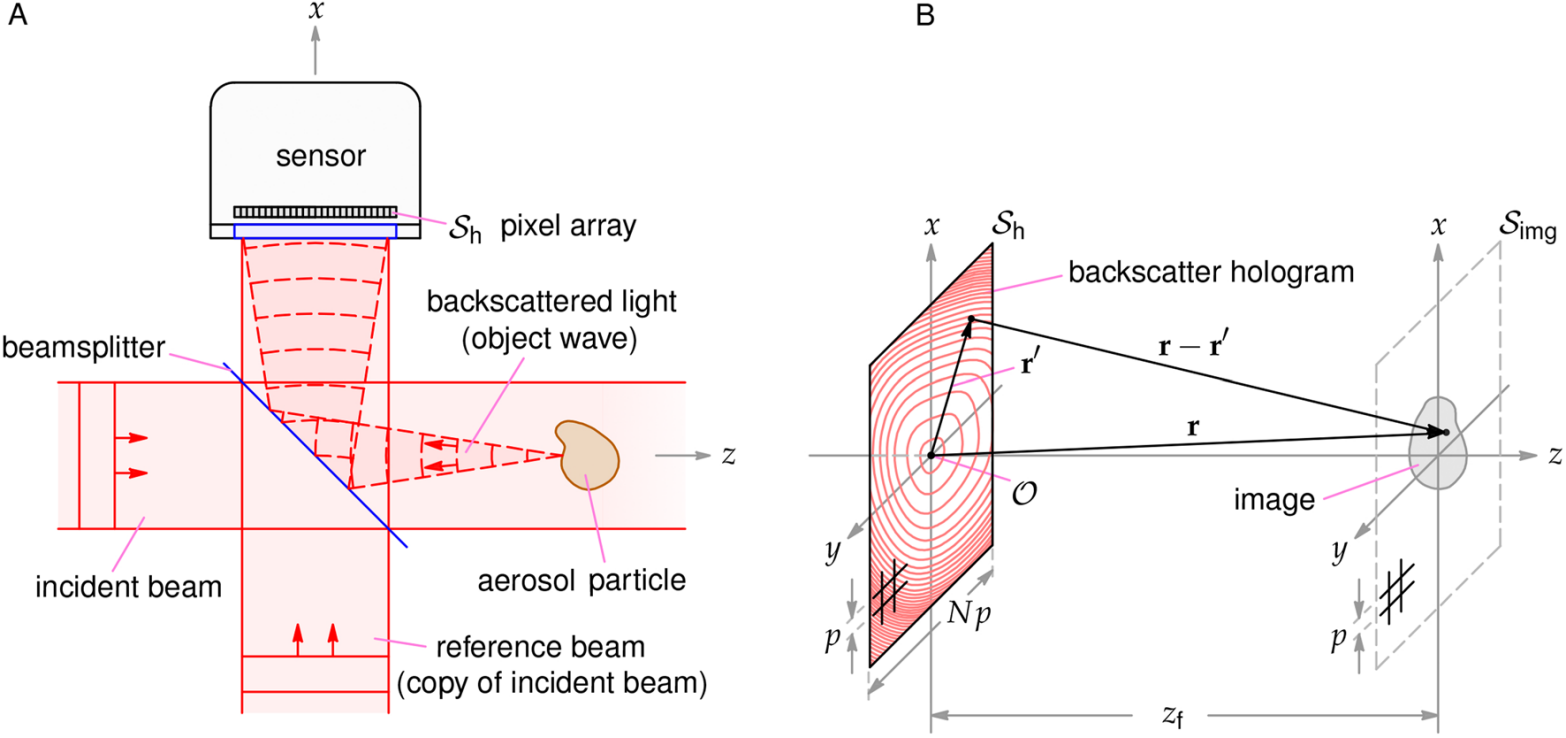
Backscatter DIH measurement and imaging principle for a single wavelength. The measurement geometry is shown in (**A**), where an aerosol particle is illuminated by an incident beam propagating along the positive *z*-axis. Backscattered light from the particle is reflected toward a sensor by a beamsplitter where it is combined with the reference beam propagating along the *x*-axis. This beam is a copy of the incident beam and originates from one half of a Mach–Zehnder interferometer as described further in the “Methods” section. The sensor’s pixel array $S_{\text {h}}$ records the interference pattern produced by the reference beam and the particle’s backscattered light, i.e., the backscattered hologram. The reflected portion of the incident and reference beams and the transmitted portion of the backscattered light at the beamsplitter do not affect the measurement and are not shown for clarity. The image reconstruction geometry is shown in (**B**). Given the beamsplitter in (**A**), the hologram is envisioned in the *x*–*y* plane and Fresnel diffraction theory^52^ is applied to reconstruct the image in the $S_{\text {img}}$ plane. The various parameters needed to implement the reconstruction process in the “Methods”section are defined in (**B**).

To explain the principle of backscatter MWDH, consider Fig. 1A where an aerosol particle is illuminated by an expanded beam, the incident beam, propagating along the positive *z*-axis. A small portion of the beam backscatters from a particle and propagates along the negative *z*-axis toward a beamsplitter where some of the light is reflected along the positive *x*-axis toward a sensor’s pixel array, $S_{\text {h}}$. Meanwhile, a copy of the incident beam from a Mach-Zehnder interferometer propagates along the *x*-axis, a portion of which passes through the beamsplitter to reach the sensor and constitute the reference beam. The object and reference light interfere across $S_{\text {h}}$ and a backscatter hologram is recorded. Suppose that this is done with only a single-wavelength for now, e.g., red light of wavelength $\lambda _{\text {r}}$ as depicted in Fig. 1, and denote the recorded hologram as $I^{\text {holo}}_{\text {r}}$. The measurement is then repeated in the absence of the the particle, resulting in a reference measurement, $I^{\text {ref}}_{\text {r}}$, which is simply the reference beam’s profile across $S_{\text {h}}$. Lastly, one forms the normalized contrast hologram as $H^{\text {con}}_{\text {r}}=(I^{\text {ref}}_{\text {r}}-I^{\text {holo}}_{\text {r}})/I^{\text {ref}}_{\text {r}}$. In doing so, background noise, such as stray light and sensor noise, is largely canceled, improving the quality of the eventual image. By combining green and blue beams, of wavelengths $\lambda _{\text {g}}$ and $\lambda _{\text {b}}$, with the red beams in a co-propagating fashion and employing a color sensor, the corresponding contrast holograms $H^{\text {con}}_{\text {g}}$ and $H^{\text {con}}_{\text {b}}$ can be recorded simultaneously.

Generally, the intensity of backscattered light from a particle is significantly weaker than forward-scattered light. For example, measurements in Sorensen et al.^47^ show that the ratio of backscattered to forward-scattered light from aerosolized Arizona road dust is as small as $10^{-4}$ and is typically $10^{-2}$ for a broader variety of particles. While not impossible to measure, the weak intensity often requires the use of amplified sensors and careful control of particle motion in a sample stream^48^ or even trapping of the particles^49,50^. Fortunately, the interference of the backscattered object wave with the reference wave effectively amplifies the signal at the sensor and such controls are thus not necessary here.

An image is then rendered from each of the holograms, $H^{\text {con}}_{\text {r}}$, $H^{\text {con}}_{\text {g}}$ and $H^{\text {con}}_{\text {b}}$, using the principles of scalar diffraction theory^26,51,52^. Conceptually, a hologram is envisioned as a transmission diffraction grating illuminated by a plane wave representing the incident beam used to record the hologram. Diffraction of the wave through the hologram converges to ultimately form the image in the $S_{\text {img}}$ plane in Fig. 1B. Here, the paraxial approximation and Fresnel transform are used to approximately evaluate the diffracted wave-amplitude at each wavelength, $E^{\text {diff}}_{\text {i}}(x,y,z)$, where the subscript $\text {i}$ indicates the wavelength, e.g., $\text {i}=1$ denotes $\lambda _{\text {r}}$, $\text {i}=2$ is $\lambda _{\text {g}}$, etc. Applying diffraction theory in this way is referred to as forward or backward propagation and further details of the evaluation are given below. An image layer is obtained by evaluating the corresponding intensity as

1 $$\begin{aligned} I^{\text {img}}_{\text {i}}(x,y)= \frac{1}{2}\sqrt{\frac{\varepsilon _{\text {o}}}{\mu _{\text {o}}}} \left| E^{\text {diff}}_{\text {i}}(x,y,z_{\text {f}})\right| ^{2}, \end{aligned}$$

where $\varepsilon _{\text {o}}$ and $\mu _{\text {o}}$ are the permittivity and permeability of free space, respectively, which are set to one for simplicity. In Eq. (1), it is necessary to know the so-called focus distance, $z_{\text {f}}$, which is approximately the physical distance *d* between the particle and the sensor in the measurement (considering reflection by the beamsplitter). Due to the particle motion, the distance is usually unknown, but may be found using an auto-focus procedure^51,52^. Finally, by combining the images for each wavelength resulting from Eq. (1) via color addition, an image is obtained approximately conveying the appearance of the particle under white light epi-illumination. Further detail for the procedure is given in Giri et al.^53^ and the “Methods” section below.

As a comment, images produced by conventional forward-scatter in-line holography exhibit an unwanted noise feature known as the twin image. In short, the in-line configuration results in the formation of both a real and virtual image of a particle during the reconstruction process. When one of these images is brought into focus, the other remains but is unfocused and usually manifests as a collection of nested rings centered on the focused image. Another source of noise is the so-called DC-term, which is essentially the image of the reference-beam profile superposed on the particle image. As explained in the many texts on the topic, e.g., in Berg^52^, these image-noise effects are strongly suppressed by the fact that the particle is much smaller than the reference-beam diameter and because the image reconstruction is done using the contrast hologram $H^{\text {con}}$ rather than the raw hologram $I^{\text {holo}}$. In this work, where the hologram is formed from backscattered light, the noise features are also present because the reference and object beams still share the same axis of propagation via the beamsplitter in Fig. 1A. And, here too, the noise features are mostly suppressed due to the particle size and use of the contrast holograms in reconstruction.

**Figure 2:**
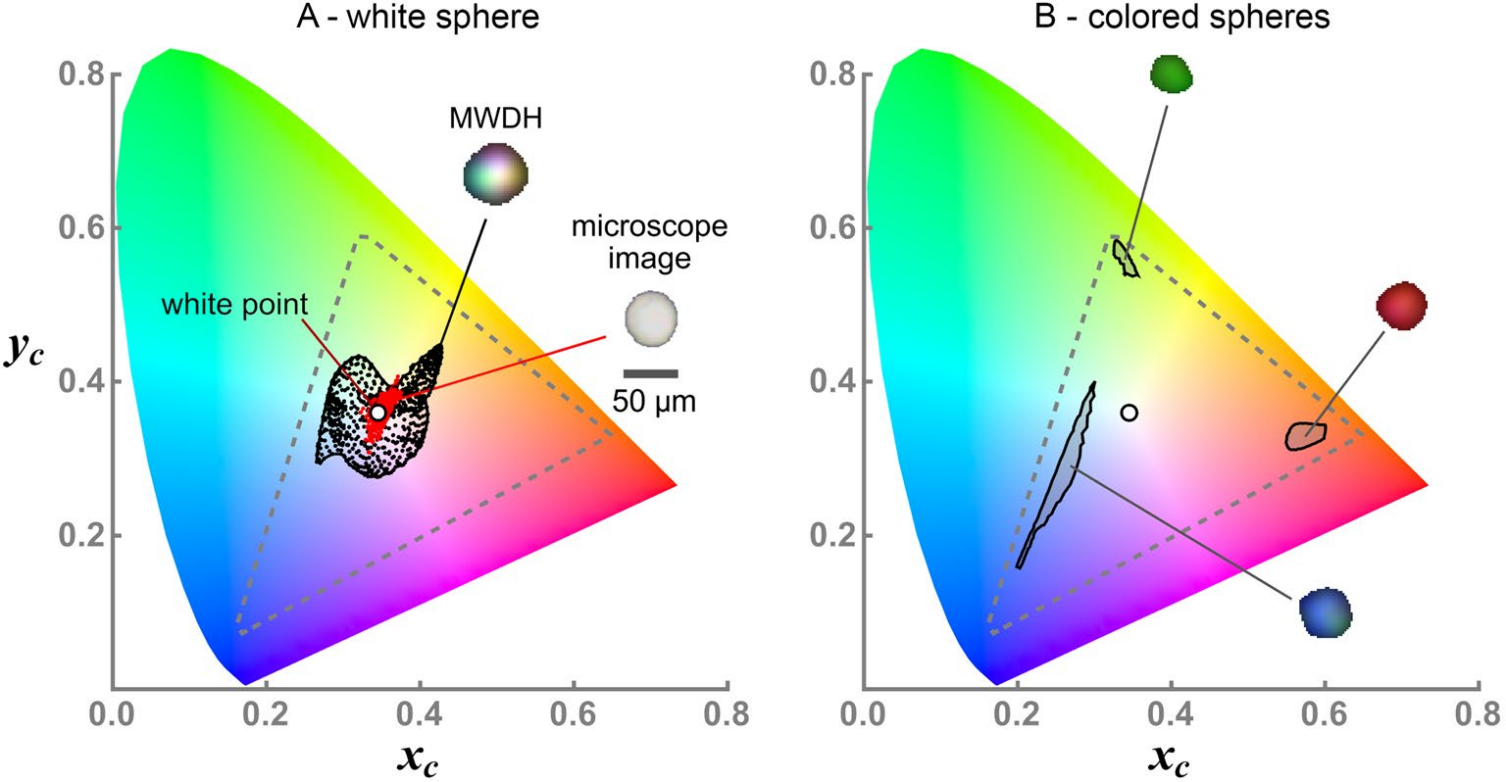
Chromaticity analysis (CA) of backscatter MWDH images of free-flowing, spherical aerosol particles. A single white aerosol particle is considered in (**A**) where the MWDH and optical microscope images are shown inset with a $50\,\upmu$m scale bar. For each image, the chromaticity points $(x_{\text {c}},y_{\text {c}})$ for each image-pixel are plotted in the color space for the MWDH image (plack points) and microscope image (red points). The sensor color-space is shown by the dashed triangular region. A contour around the point-groups defines the chromaticity signature and both signatures surround the color-space white point indicating an overall white appearance of the particle image. Note that the microscope image is taken for a representative particle from the stock powder from which the aerosol is generated. So, this image does not show the same particle as the aerosol (MWDH) image. In (**B**) is shown the MWDH images of single red, green, and turquoise spherical aerosol particles and their corresponding chromaticity signatures. Due to the relatively pure-color appearance of each particle, their corresponding signatures are isolated near the corners of the color space. Notice that the colors displayed in the particle images here appear darker than the associated colors outlined in the chromaticity plot. Recall, however, that CA does not depend on the brightness of a given color (see “Methods” section), and hence, the same colors are being represented.

Given that the aim is to the use the color images to realize a basic material-differentiation capability, a method is needed to objectively quantify the image color-content. This is achieved with chromaticity analysis (CA), which in short, represents the color of each pixel in an image as a point $(x_{\text {c}},y_{\text {c}})$ in a color space^54^, see the “Methods” section. The color space encompasses the range of colors resolved by the sensor, including a unique point, the white point, where the colors combine to yield white. In this work, the common sRGB color space is used^55^. An example is shown in Fig. 2A where CA is presented for the backscatter MWDH image of a single $50\,\upmu$m diameter white spherical aerosol particle. The chromaticity points corresponding to the MWDH image are shown in black while those for a conventional white-light microscope image in epi-illumination mode are shown in red. The points cluster around the white point, indicating an overall white appearance in either image. The closer the points are to the white point, the more purely white the image is, revealing that the microscope image has superior color rendition compared to the backscatter MWDH image. The reason for this difference is that MWDH is a coherent imaging process, and thus, suffers from speckle noise in each color channel^53^. More discussion on this point and a description for the measurement will follow.

The discrimination of colors between different images is presented in Fig. 2B. Here, MWDH images of a red, green, and turquoise spherical aerosol particle are shown along with their chromaticity points. Instead of plotting each point, a contour is shown outlining the boundary of the point group; this contour will constitute the image’s chromaticity signature in the following. It is evident here that the particles, with distinctly different colors, yield signatures that are clearly distinguishable in the color space. Of course, this distinction is trivial from the visual appearance of the images. One will see, however, that more subtle differences in color can occur, which are better represented by the signatures rather than a subjective assessment by-eye.

## Results

**Figure 3:**
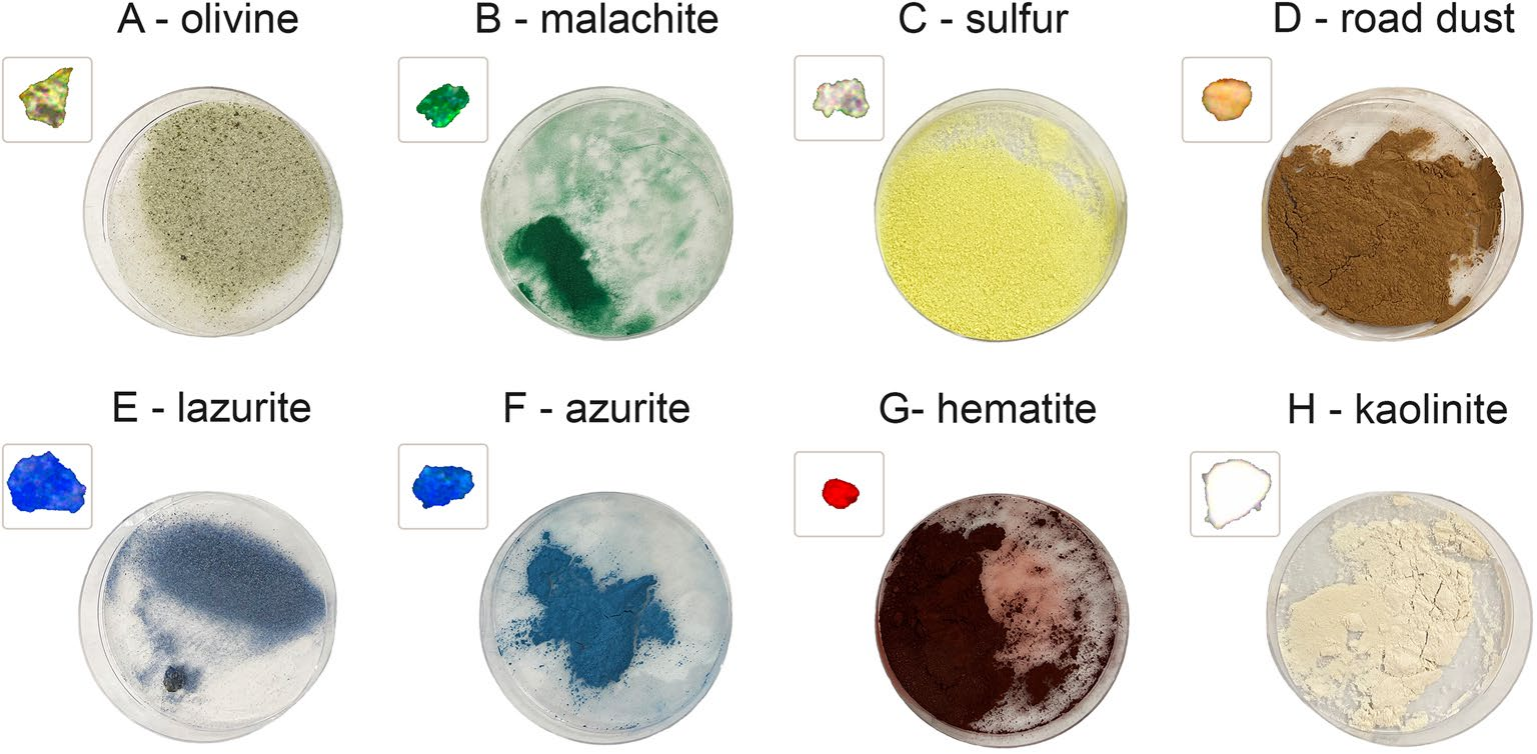
Photograph of the mineral-dust stock powders used to produce the aerosols in Fig. 4. The powders result from pulverizing various rock samples followed by sieving to retain particles only in the $40-210\,\upmu$m size range as mentioned in the “Methods” section. Shown beside each powder is a microscope image of a single particle from the powder. See Fig. 4 for a scale bar to estimate the size of the microscope images.

To demonstrate the performance of backscatter MWDH, a variety of mineral dust particles are studied that exhibit vivid colors. They include olivine, malachite, sulfur, road dust, lazurite, azurite, hematite, and kaolinite. The minerals are chosen because most display a roughly single color, e.g., the red of hematite, and can thus serve as a test for the concept that material differentiation may be achieved via the chromaticity signatures. Figure 3 shows the dust sample powders, which are generated via the procedure discussed in the “Methods” section. Here, a microscope image of a single particle from the powder is shown adjacent to the image of the powder itself. To obtain the microscope image, a small amount of powder is dispersed across a microscope slide and imaged in epi-illumination mode under white-light illumination. Thus, the image is formed from reflected light akin to the later MWDH experiments. It is perhaps interesting to note that the colors displayed by an individual particle do not necessarily correspond to the visual appearance of the powder. One explanation for such discrepancy is the fact that if absorption is a significant factor accounting for a powder’s color, that same color may not manifest as pronounced at the single-particle scale due to the comparatively small volume of the particle material compared to that of the powder sample. Moreover, there is usually some degree of particle-to-particle variability in appearance. What one sees for the powder is better regarded as an average color and brightness produced by the many constituent particles.

**Figure 4:**
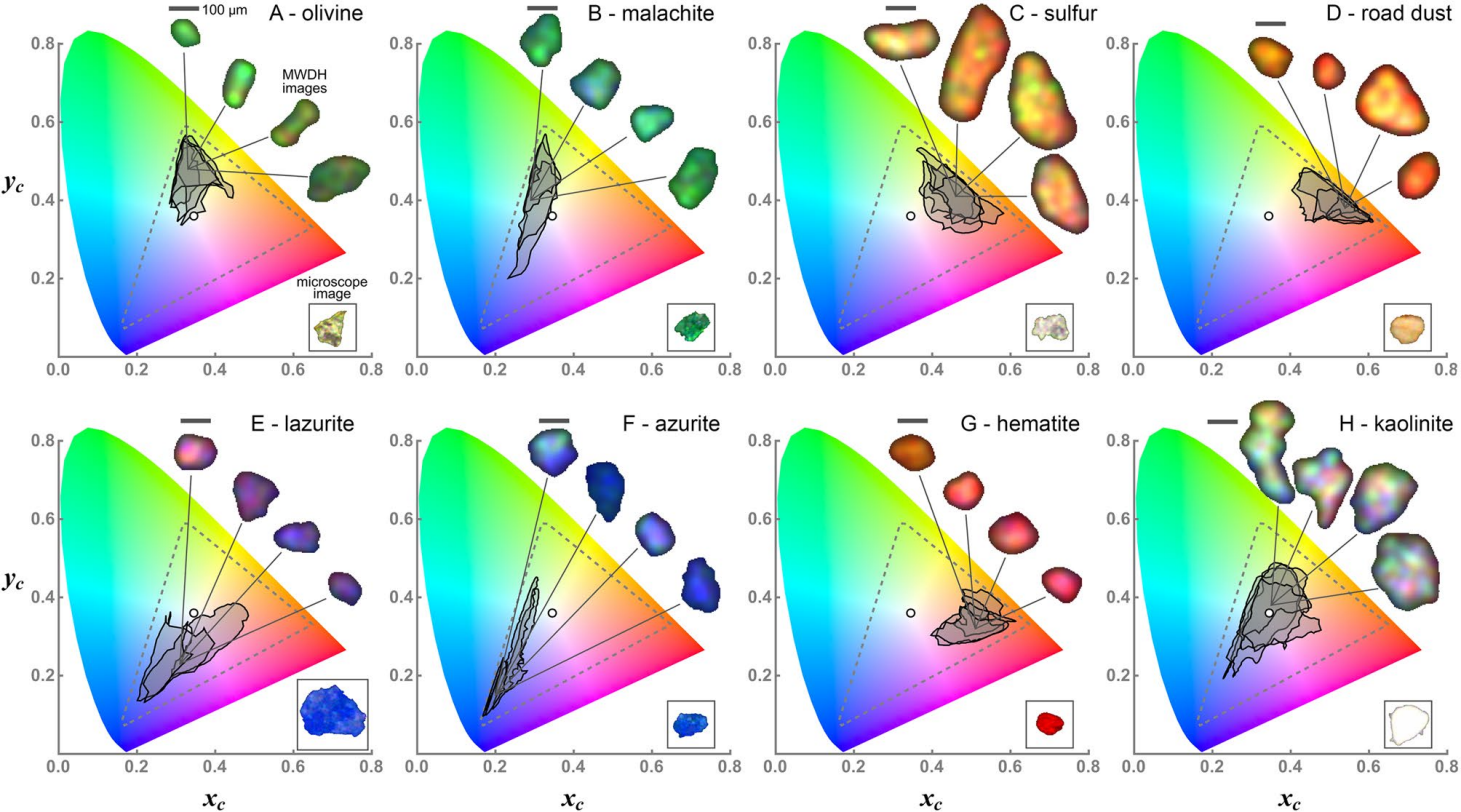
Chromaticity analysis of a variety of free-flowing mineral dust aerosol particles. Each panel shows the backscatter MWDH image of the particles associated with their chromaticity signatures. Each particle-image corresponds to a separate measurement. A $100\,\upmu$m scale bar is shown along with an optical microscope image, in frame, of a representative particle taken from the stock powder from which the aerosols are generated, see also Fig. 3.

Figure 4 presents CA of the backscatter MWDH images of the mineral dust aerosols. First consider Fig. 4A for olivine. This mineral is generally light yellow-green in appearance, which can be seen in the framed inset image of Fig. 4A or Fig. 3A. The other insets in Fig. 4A are the backscatter MWDH images of individual aerosol particles that trigger hologram measurements at various times in the experiment. The chromaticity signatures of these images are shown, being clearly in the yellow–green portion of the color space with much of their signatures overlapping. The overlap indicates that the images possess similar color content despite the variation in overall brightness of the images shown.

Malachite is shown in Fig. 4B, where again we see overlap of the signatures, mostly green, but also some variability indicating that the images exhibit a degree of blue content. Progressing though the other materials in Fig. 4, one sees the aerosol signatures continue to evolve in their location in the color space. Overall, the backscatter MWDH images display a similar color content as the microscope images, although with notable exceptions. For example, the kaolinite images in Fig. 4H show a comparatively large signature surrounding the white point. This indicates a greater variety of colors than what is displayed by the other minerals, which is evident from the multicolored marbled appearance of the MWDH images. Meanwhile, the microscope image reveals a uniformly white appearance. The additional colors seen in Fig. 4H are due to speckle noise caused by the relatively stronger backscatter in each color by this material. Other materials, such as the hematite in Fig. 4G absorb the green and blue wavelengths well, producing significant backscattering only for red light and speckle noise is thus significant only in the red image-layer. Similar behavior is seen with the sulfur particle in Fig. 4C, where speckle is most evident in the red and green layers.

Comparing the MWDH and microscope images in Fig. 4 shows that the former appear smoother in shape than the latter. This too is a consequence of speckle. As described below and in Giri et al.^53^ and Bianco et al.^56^, the MWDH images are convolved with a weak Gaussian filter to partly suppress the effect of speckle, and in doing so, a degree of resolution is lost. Nevertheless, the nonspherical nature of these particles remains evident. The size $\eta$ of the speckle grains varies with $\lambda$ and *d* and can be estimated from Blanche^26^. For this work, $\eta _{\text {i}}$ ranges from approximately 16 to $30\,\upmu$m, which effectively sets the best image resolution that can be achieved. It is for this reason that the resolution is poorer than the forward-scatter DIH images of similar aerosol particles using comparable optical designs, e.g., see Berg et al.^31^ or Kemppinen et al.^45^. So, the effect of speckle and the limited number of laser wavelengths used prevent the MWDH images from having the same quality of color rendition and image resolution that is obtained by a microscope image. Yet, it is not the objective of this work to reproduce the image quality of a microscope. The intent is to test whether color in holography can be useful to differentiate aerosol particles of different materials, and Fig. 4 demonstrates that it is, at least for the materials considered here.

**Figure 5:**
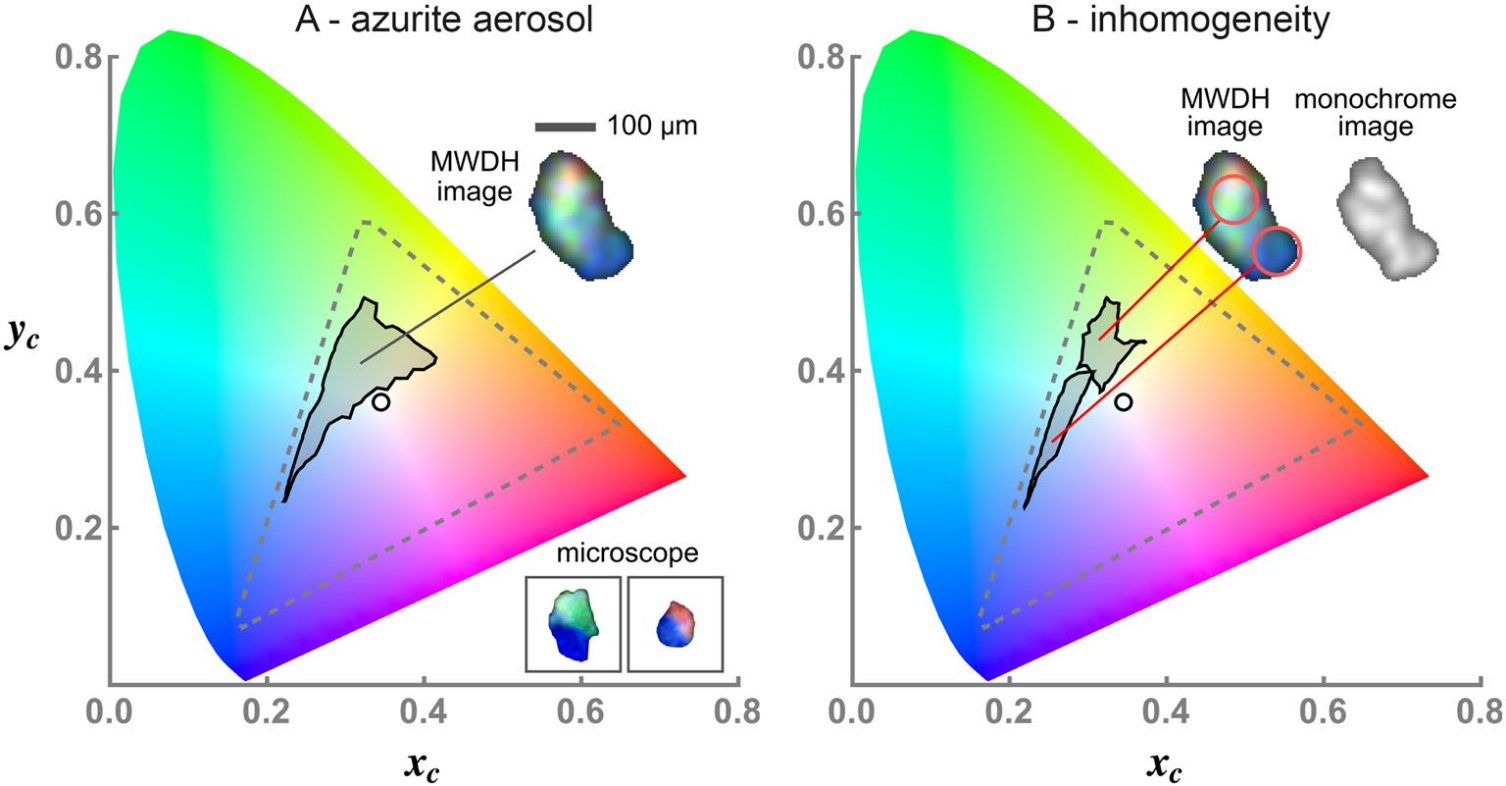
Possible observation of material inhomogeneity within a single mineral-dust aerosol particle. The chromaticity signature of an azurite particle is shown in (**A**), displaying both green and blue contributions. In the MWDH image, the particle shows distinct portions of green or blue that are significantly larger than the $\sim 30\,\upmu$m speckle size. This suggests that the particle is inhomogeneous, being largely composed of a green and blue mineral. A smaller red component is also visible. The microscope images of particles taken from the stock powder used to generate the aerosol, see Fig. 3, also display this inhomogeneity in some cases. The same aerosol particle is considered in B, except the chromaticity signature is plotted only for the particle-image pixels contained within the circles shown. The blue signature appears in the same general area of the color space as the azurite particles in Fig. 4F, while the green signature best corresponds to the malachite in Fig. 4B. This suggests that the particle is a combination of malachite and azurite, which is known to occur for this mineral^57^. The other image in (**B**) displays the result of reconstructing the same particle image using only $\lambda _{\text {b}}$, producing a monochrome image. No clear evidence of the inhomogeneity is seen.

Given that $\eta _{\text {i}}$ is significantly less than many of the particle sizes in Fig. 4, it may be possible to resolve material inhomogeneity within a single aerosol particle in some cases. That is, if a particle’s MWDH image were to display different colors in regions of the image that are significantly larger than the speckle, then it is reasonable to conclude that such regions are composed of different materials. The mineral azurite, a basic copper carbonate, is a good example. Consider the microscope images of the two particles in Fig. 5A shown in frames. An inhomogeneous composition is clearly seen from the blue and green or blue and pink components. Azurite, which is $\text {Cu}_{3}(\text {CO}_{3})_{2}(\text {OH})_{2}$ and blue in appearance, is often found in combination with malachite, which is $\text {Cu}_{2}(\text {CO}_{3})(\text {OH})_{2}$ and green in appearance^57^. The appearance of the MWDH image in Fig. 5A is unlike the blue appearance of the particles in Fig. 4F in that it shows large singular green and blue regions across the image, which is most clear from the chromaticity signature. Given the size of either region is two or three times larger than the speckle, it is likely that this particle is similar in composition to the left-most microscope image in Fig. 5A. If so, this means that the MWDH image is able to resolve the inhomogeneous composition, viz., the malachite (green region) and the neighboring azurite (blue region). The small red-colored region in the MWDH image in Fig. 5A could suggest a third component similar to the other microscope image shown. Such color could be due to the presence of cuprite $\text {Cu}_{2}\text {O}$ in the mineral, which has a red appearance; of course, it could also be a random dominate red-speckle as well.

To further illustrate how backscatter MWDH resolves the material inhomogeneity, consider Fig. 5B. Rather than plotting the chromaticity signature for the whole image, the signatures for pixels in the two circles in the image are shown. Comparing these two signatures to those in Fig. 4 shows that the blue signature resides generally in the same portion of the color space as those for the azurite in Fig. 4F while the green signature is similar to those of malachite in Fig. 4B. In other words, provided the particle is sufficiently larger than the speckle, it is possible to scan a window over the image (the circle here) looking at the corresponding signatures and then propose associations to a library of signatures of known materials, e.g., Fig. 4, to classify the particle composition and possible inhomogeneity. While it would be difficult to prove a specific identification of material in this manner, there is often supplementary information available that could narrow the possibilities. For example, one may know that (yellow) pollen is not expected in a particular geographical region, such as a desert, and so the observation of yellow particles could be associated with mineral dust.

A natural question would be whether the inhomogeneity in Fig. 5 could be resolved with the use of a single wavelength, i.e., monochrome backscatter DIH. To test this, the image is reconstructed in Fig. 5B using only the blue wavelength. The result is an image that is almost identical to the MWDH image in terms of overall size and shape. Yet, there is little variation of gray-scale density across the image, which would be essentially the only way to infer any inhomogeneity in this case. This observation thus underscores the utility of backscatter holographic imaging using light at multiple wavelengths. As a final comment, note that the size of the circles used in Fig. 5B are approximately twice the speckle. This could cast doubt as to whether a color-based resolution of particle inhomogeneity is really being seen, or if it is simply a speckle effect. The key point, however, is that continuous portions of the particle significantly larger than the speckle, and indeed larger than the circles, display a roughly similar color, i.e., the green and blue regions.

## Methods

### Experiment

**Figure 6:**
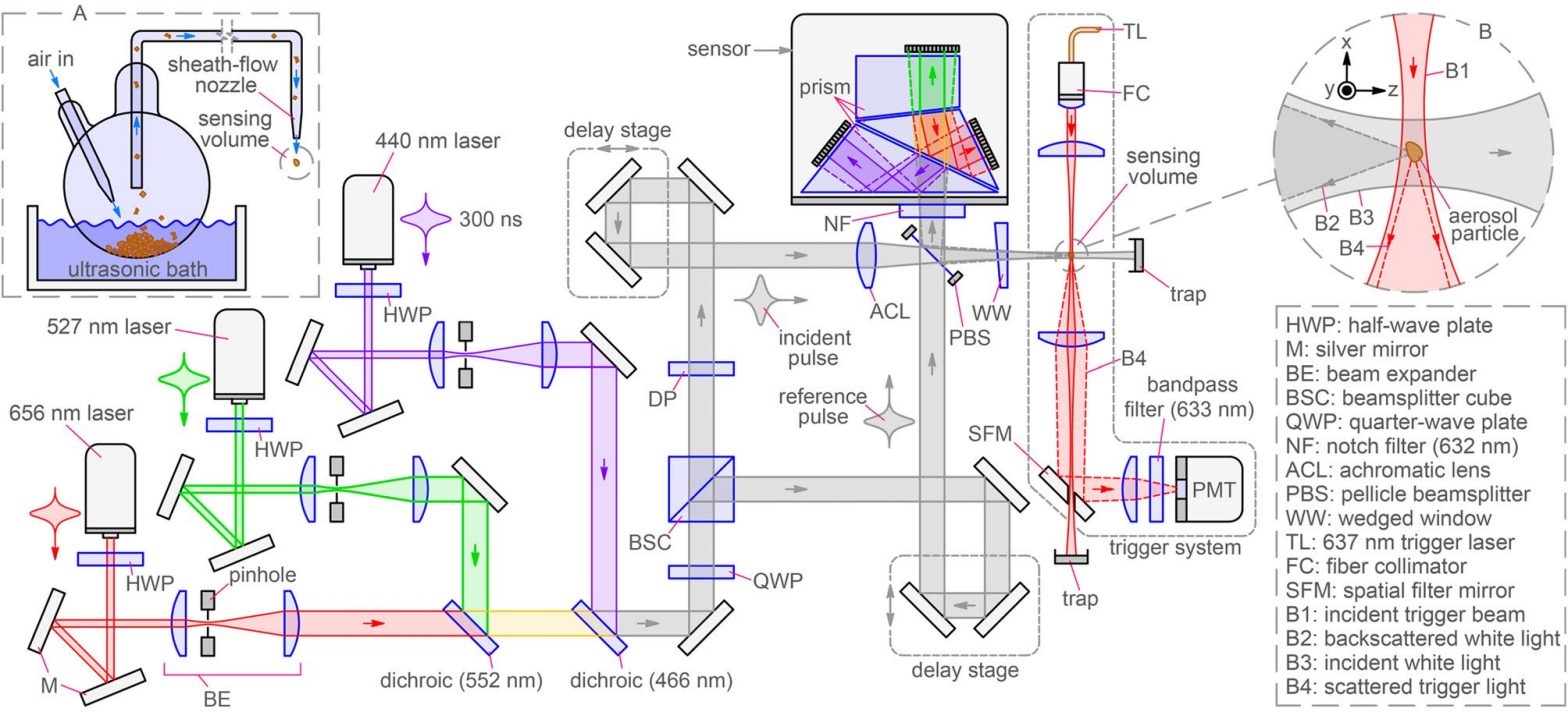
Optical design used to record color backscatter-holograms of free-flowing aerosol particles. Pulsed red ($\lambda _{\text {r}}=656$ nm), green ($\lambda _{\text {g}}=527$ nm), and blue ($\lambda _{\text {b}}=440$ nm) laser beams are combined into a white beam, which is split by the BSC to form incident and reference beams. A Mach-Zehender configuration is used to interfere the light backscattered by an aerosol particle in the sensing volume with the reference beam at the sensor. The particles are delivered to the sensing volume via a sheath-flow nozzle fed by an aerosol generator, shown in (**A**). An optical trigger system senses when a particle is present in the volume and supplies a signal to activate emission of a 300 ns pulse from each laser and begins the sensor exposure. This is achieved by illuminating the sensing volume by a CW trigger beam (B1) in inset (**B**), which scatteres from any aerosol particle present. The scattered light (B4) is separated from unscattered light by the mirror SFM and then sensed by the PMT to create the trigger signal. The coordinate system is shown in inset (**B**) where the incident (white) holography beam (B3) defines the positive *z*-axis and the backscattered light in (**B**) is labeled (B2).

The backscatter MWDH optical design is based on Giri et al.^53^ where color holographic images of *stationary* particles are achieved. To apply the concept to aerosols, however, involves a number of challenging adaptations. Due to the particle motion, the lasers must be pulsed to prevent the motion from washing-out the hologram fringes resolved by the sensor. The pulses must also be synchronized and coordinated to emit only when a particle is present in the sensing region. Compared to the CW lasers used in Giri et al.^53^, the pulsed nature of the lasers reduces the coherence length $\ell _{\text {c}}$ to approximately $\ell _{\text {c}}= 1-2$ mm, as measured by a Michelson interferometer and estimated from the spectral width of each laser. With conventional forward-scatter DIH, the reduced $\ell _{\text {c}}$ would not be problematic as the reference and object waves co-propagate to the sensor. For backscatter DIH, however, a portion of the beam must be split-off to serve as the reference wave, which is then combined with the backscattered light in a Mach-Zehnder configuration as shown in Fig. 6. Thus, the path length of the arms of the interferometer must be equal to within $\ell _{\text {c}}$, which is achieved by delay stages.

Three diode-pumped solid state (DPSS) lasers emit triggered pulses of pulse-length $\tau \sim 300$ ns. The red laser (CrystaLaser LLC, QL-656-100) has a wavelength of $\lambda _{\text {r}}=656$ nm and a pulse energy of ${\mathscr {E}}_{\text {r}}=15\,\upmu$J, the green laser (Photonics Industries International, Inc., DC-50-527) has $\lambda _{\text {g}}=526$ nm with ${\mathscr {E}}_{\text {g}}=1.0$ mJ, and the blue laser (CrystaLaser LLC, QL-440-50) has $\lambda _{\text {b}}=440$ nm with ${\mathscr {E}}_{\text {b}}=10\,\upmu$J. Each beam is passed through a half-wave plate (HWP) to align the (linear) polarization of the red and blue beam to be parallel to the table and the green beam vertical. The beams are cleaned and expanded to approximately 5 mm in diameter by beam expanders (BE) and then combined via dichroic beamsplitters (IDEX Health & Science LLC/Semrock, LM01-552-25 and LM01-466-25) with 552 nm and 466 nm cut-on wavelengths. The procedure generates a nearly white beam.

The white beam is passed through an achromatic quarter-wave plate (QWP: Thorlabs Inc., AHWP05M-580) and is then split by a beamsplitter cube (BSC) with a transmission-to-reflection ratio of 70% to 30% to form the incident and reference beams, respectively. The incident beam, propagating vertically in Fig. 6 from the BSC, passes through a depolarizer (DP: Thorlabs Inc., DPP25-A), which partly suppresses speckle in the eventual image. Next, the beam transits a delay stage and is focused to a waist by an achromatic lens (ACL: Thorlabs Inc., AC254-150-A) into the sensing volume where it illuminates an aerosol particle. The positive *z*-axis is defined by this beam (B3) as shown in the Fig. 6B inset. Backscattering from small particles is usually far less intense than forward, or side, scattering. By bringing the incident beam to a waist near the particle, the intensity is significantly greater than if a collimated beam were used, and thus, the backscattered light is also more intense. Meanwhile, the other beam from the BSC, propagating to the right from the BSC in Fig. 6, enters a delay stage and is then directed into the sensor. This beam constitutes the reference wave.

The object wave is the light backscattered by a particle in the sensing volume and propagates along the negative *z*-axis, see B2 in the Fig. 6B inset. A pellicle beamsplitter (PBS: Thorlabs Inc., BP145B1) is used to direct this light into the sensor where it interferes with the reference wave. The resulting holograms, $I^{\text {holo}}_{\text {r}}$, $I^{\text {holo}}_{\text {g}}$, and $I^{\text {holo}}_{\text {b}}$ are resolved by the three global-shutter CMOS arrays in the sensor (JAI Inc., AP-3200T-PGE). Here, the white light of the combined reference and object waves is separated into its red, green, and blue components by coatings on the sensor’s prisms. While not required, use of this type of sensor is far superior to a single-CMOS or CCD sensor with a Bayer filter as so-called cross-talk between the color channels is nearly eliminated. The CMOS arrays (Sony, IMX265) have pixel size $p=3.45\,\upmu$m and an array size of $2064\times 1544$ pixels. The specific laser wavelengths $\lambda _{\text {r}}$, $\lambda _{\text {g}}$, and $\lambda _{\text {b}}$ are chosen to reside in the red, green, and blue (RGB) channels of the sensor, further details for which can be found in Fig. 1(a) of Giri et al.^53^.

An optical trigger system determines when a particle is passing through the sensing volume. The system involves a $\lambda =637$ nm CW beam from a fiber pigtailed DPSS laser (Coherent Inc., OBIS-LX-100-FP) propagating along the negative *x*-axis that is focused to a waist in the sensing volume. The beam is perpendicular to the white holography beam in the volume. A particle passing through the trigger beam will scatter a portion of the light, which is collimated by another lens. The scattered light is then reflected by a mirror (SFM) to a photomultiplier tube sensor (PMT: Hamamatsu Photonics, H6780-20) while the intense unscattered portion of the beam is focused through a $500\,\upmu$m diameter hole in that mirror (Lenox Laser Inc., AL-45-500) and discarded in a beam trap. In this way, a transient in the PMT voltage indicates an aerosol particle is present in the sensing volume and serves as a master trigger signal to activate the sensor and three holography lasers. There is a delay of $159\,\upmu$s between the receipt of a trigger signal and when light-integration begins in the sensor. Thus, if the lasers were triggered by the master signal, no light would be captured by the sensor due to the latency. To resolve the issue, a digital delay generator (Stanford Research Systems Inc., DG645) is used to add a delay between the master signal and the triggers sent to the lasers.

Given the weak intensity of the backscattered light, stray light becomes a significant concern. For example, scattered trigger light must be prevented from reaching the sensor or $I^{\text {holo}}_{\text {r}}$ could be affected. To do so, the sensor is guarded by a notch filter (NF: IDEX Health & Science LLC/Semrock, NF03-633-25) that blocks only the trigger light. A bandpass filter (Thorlabs Inc., FL05635-10) likewise prevents any stray holography light from reaching the PMT. Although all the optical elements, other than mirrors, are anti-reflection (AR) coated, there is still an amount of stray light originating from multiple reflection between the various optical surfaces. The impact of such light at the sensor is minimized by AR-coated wedged windows (WW: Thorlabs Inc., WW41050-A) at multiple places in the layout, one of which is shown in Fig. 6.

Recall that the optical path-length of the arms of the Mach-Zehender must be matched to within the coherence length $\ell _{\text {c}}$ of the holography lasers. The path-length of the incident-beam arm begins at the BSC, then runs through the delay stage, then along the positive *z*-axis to the sensing volume, and finally back along the negative *z*-axis to the PBS. The reference-beam arm begins at the BSC, then runs through its delay stage, and finally along the positive *x*-axis to the PBS. To match these path-lengths, a bare glass fiber is placed where the aerosol stream would otherwise flow in the sensing region. The delay stages are adjusted until a backscatter hologram of the stationary fiber appears on the sensor. The fiber is then removed and the aerosol measurements may be conducted.

### Image generation and processing

**Figure 7:**
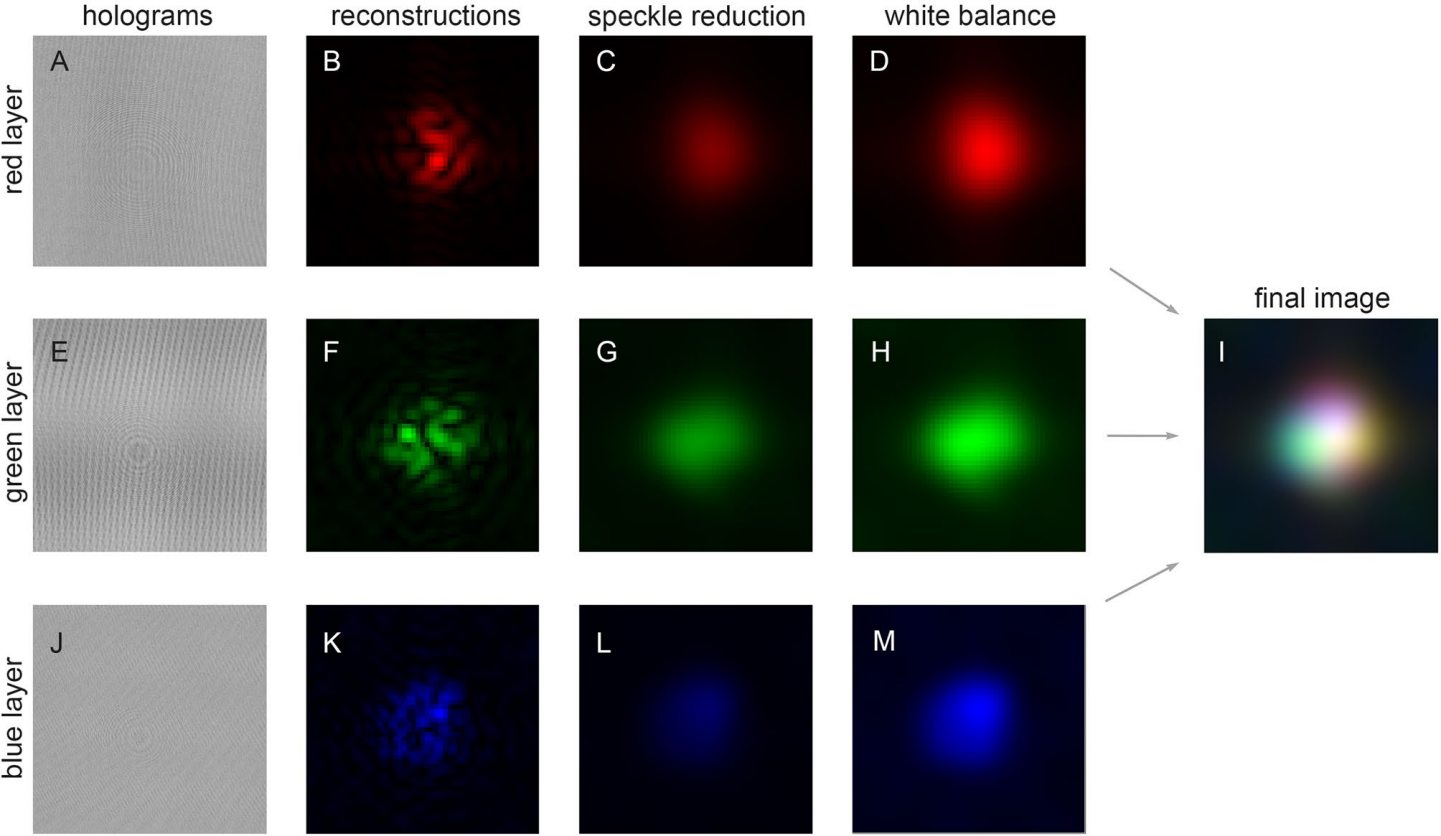
Backscatter MWDH image-generation process. The backscatter contrast holograms $H_{\text {i}}^{\text {con}}$ measured for a $50\,\upmu \text {m}$ white spherical aerosol particle are shown in the first column (**A,E,J**). This particle establishes the WB and is the same particle presented in Fig. 2A. Note that the interference fringes attributed to the particle are difficult to see due to the weak intensity of the backscattered light. Close examination of the holograms shows a faint cluster of nested circular fringes, which are due to the particle, and the most visible example is near the center of hologram (**E**). The second column (**B,F,K**), shows the image layers reconstructed from these holograms, where speckle is evident. Applying the speckle-reduction process yields the images in the third column (**C,G,L**), which are then white-balanced to give the images in the forth column (**D,H,M**). The three processed image-layers (**D,H,M**), are then combined via color addition to give the final particle image shown in (**I**).

Once the three-color backscatter holograms of a particle are recorded, an identical measurement is done with no aerosol present to record reference measurements in each color channel, i.e., $I^{\text {ref}}_{\text {r}}$, $I^{\text {ref}}_{\text {g}}$, and $I^{\text {ref}}_{\text {b}}$. From these, the three contrast holograms $H^{\text {con}}_{\text {r}}$, $H^{\text {con}}_{\text {g}}$, and $H^{\text {con}}_{\text {b}}$ mentioned above can be formed. For reference, the contrast holograms for the white spherical aerosol particle of Fig. 2A are presented below in Fig. 7 ($1{\text {st}}$ column). Single-color, i.e., monochrome, images $I^{\text {img}}_{\text {i}}$ of the particle in each color $(\text {i})$ are then reconstructed by computing the back-propagated diffracted wave amplitude $E^{\text {diff}}_{\text {i}}$ as follows. Each $H^{\text {con}}_{\text {i}}$ is cropped to be a square array of $N\times N$ pixels with the particle’s nested-ring interference pattern near the array center. Note that the pattern is usually not visible in the raw holograms, $I^{\text {holo}}_{\text {r}}$ etc., due to the weak intensity of the backscattered wave, but it does usually appear in the contrast hologram, see Fig. 7. Next, the shifted impulse-response function $h^{\text {sh}}_{2N,\text {i}}$ is calculated in an array of size $2N\times 2N$ as

2 $$\begin{aligned} \begin{aligned} h_{2N,\text {i}}^{\text {sh}}&(j,k,z)=\\&\frac{p^{2}}{i\lambda _{\text {i}} z} \exp {\left( \frac{2\pi i z}{\lambda _{\text {i}}}\right) } \exp {\left\{ \frac{i\pi p^{2}}{\lambda _{\text {i}} z} \left[ \left( j-N\right) ^{2}+ \left( k-N\right) ^{2}\right] \right\} }, \end{aligned} \end{aligned}$$

where *j* and *k* are indexes that range in $1\le j \le 2N$ and $1\le j \le 2N$. The contrast holograms are likewise shifted and also zero-padded^52^ to the same array size, i.e., $H^{\text {con}}_{2N,\text {i}}$ as

3 $$\begin{aligned} H^{\text {con}}_{2N,\text {i}}(j,k)= \left. {\left\{ \begin{array}{ll} H^{\text {con}}_{\text {i}}\left( j-\dfrac{N}{2},k-\dfrac{N}{2}\right) , &{} j\wedge k\in \left[ \dfrac{N}{2} +1,\dfrac{3N}{2}\right] , \\ 0, &{} \text {otherwise.} \end{array}\right. } \right. \end{aligned}$$

Next $E^{\text {diff}}_{\text {i}}$ is calculated using fast Fourier transforms ${\mathscr {F}}$ and their inverse $({\mathscr {F}}^{-1})$ as

4 $$\begin{aligned} \begin{aligned}{}&E^{\text {diff}}(m,n,z)=\\&E_{\text {o}}\, \text {Sh}_{N}\Bigg \{ {\mathscr {F}}^{-1}\Big \{ {\mathscr {F}}\big \{ H^{\text {con}}_{2N}(j,k)\big \}\odot {\mathscr {F}}\big \{h^{\text {sh}}_{2N}(j,k,z)\big \}\! \Big \}\!\Bigg \}, \end{aligned} \end{aligned}$$

where the symbol $\odot$ denotes element-wise multiplication and $\text {Sh}_{N}\left\{ \ldots \right\}$ signals to swap the quadrants of the $2N\times 2N$ array and then retain only the central $N\times N$ portion of the final result. Once $E^{\text {diff}}_{\text {i}}$ has been computed, the red, green, and blue image-layers $I^{\text {img}}_{\text {i}}$ are rendered via Eq. (1). Equations (1) and (4) are iteratively re-evaluated following the auto-focus algorithm of Simobaba^51^ using the Tamura coefficient until $z=z_{\text {f}}$, i.e., until the particle in each image layer is focused. Figure 7 ($2{\text {nd}}$ column) shows these reconstructed image-layers for the white spherical aerosol particle in Fig. 2A. Further details of the reconstruction operations and open-source code in the *Mathematica* language to implement them are given in Berg^52^.

Two processing steps are applied to the (single color) image-layers $I^{\text {img}}_{\text {i}}$ before they are combined into a color image: speckle reduction and white balance. Generally, the size of the speckle grains in each image layer $\eta _{\text {i}}$ is^26^

5 $$\begin{aligned} \eta _{\text {i}}= \frac{\lambda _{\text {i}}z_{\text {f}}}{Np}, \end{aligned}$$

which evaluates to approximately $\eta _{\text {r}}\sim 24\,\upmu$m, $\eta _{\text {g}}\sim 20\,\upmu$m, and $\eta _{\text {b}}\sim 16\,\upmu$m. The depolarizer (DP) in Fig. 6 partly reduces the speckle by perturbing the polarization direction across the white holography beam-profile, but it cannot eliminate the speckle. Further reduction of the speckle is achieved by convolving each image layer with a Gaussian kernel^56^, i.e., Gaussian filtering (GF), as

6 $$\begin{aligned} F_{\text {i}}(x,y)= \iint _{S_{\text {img}}} \!\!\!\!\!\!\!\!I^{\text {img}}_{\text {i}}(x',y')G_{\sigma }(x-x',y-y')\, \text {d}x'\text {d}y' \end{aligned}$$

where $F_{\text {i}}$ is now the $\text {i}^{\text {th}}$ filtered image-layer, the integral is carried out over the cropped image array $S_{\text {img}}$, and the Gaussian kernel is

7 $$\begin{aligned} G_{\sigma }(x,y)= \frac{1}{2\pi \sigma ^{2}} \exp {\left[ -\left( \frac{x^{2}+y^{2}}{2\sigma ^{2}} \right) \right] }, \end{aligned}$$

where $\sigma$ is the filter’s standard deviation. Applying Eq. (6) to an image has the effect of adding uniform blur, and thus, reduces the impact of speckle at the cost of image resolution^53^. A value of $\sigma =\eta _{\text {i}}/2$ is found to be a good balance between speckle reduction and the loss of image resolution. Figure 7 ($3{\text {rd}}$ column) shows the effect of applying this speckle reduction to each image layer for the white spherical aerosol particle in Fig. 2A.

A white balance (WB) process is also required because the holography lasers differ in average power and the sensor’s spectral response is not constant from $\lambda _{\text {r}}$ to $\lambda _{\text {b}}$. The process involves capturing a contrast hologram of an aerosolized $50\,\upmu$m diameter, nonfluorescent, white polymer sphere. The image layers are reconstructed and speckle reduction is performed. The maximum pixel-value of each of the resulting layers $F_{\text {i}}$ is then found, from which the scale-factors $\alpha _{\text {i}}$ needed to bring the maximum values to one are calculated. Multiplying the factor by each pixel in $F_{\text {i}}$ ensures that any given pixel has a value ranging from 0 to 1. Figure 7 ($4{\text {th}}$ column) shows the effect of applying this WB process to the speckle-reduced image layers for the white spherical aerosol particle of Fig. 2A. Finally, the three layers are combined via color addition to yield a color image displaying a white appearance^53,58^. The result is shown in Fig. 2A and in Fig. 7I. Color addition means that first each pixel in an image layer, e.g., the red layer $F_{\text {r}}$, is assigned the color red, *R*, with a saturation between zero and one, $R\in [0,1]$. This is done for the green *G* and blue *B* layers and the three are combined into a single image where each pixel now expresses an *RGB* color. A white pixel has the color $RGB=(1,1,1)$, a black pixel has $RGB=(0,0,0)$, a pure green pixel has $RGB=(0,1,0)$, etc. The scale-factors $\alpha _{\text {i}}$ found for the white particle define the measurement’s WB and are then applied to the image layers of other particle types.

Although the intent here is not to produce color images of the particles on-par with the quality of a microscope, it is relevant to give some background for the color analysis used, viz., the CA. Color vision in humans involves three receptors, the cones, and the perception of color has a property known as trichromacy. Trichromacy is the idea that given three light sources, where each is a primary color, e.g., red, green, or blue, any other perceptible color may be rendered by a mixing of these primaries, which is also known as color addition. There is a degree of freedom in the choice of primaries. For example, they can be monochromatic, as they are here, or they can be sources with finite bandwidths. An important characteristic of the primaries is that a mixture of any two should not yield the same perceptual response, i.e., color, as the third. The color perceived can then be described by tristimulus values, labeled *X*, *Y*, and *Z* by convention^59^; they depend on the primaries and quantify the sensitivity of the eye’s cones. The *X*, *Y*, and *Z* values represent coordinates of a 3D vector in color space. The vector’s direction specifies color and its length gives the amount of color, i.e., the color’s luminance. Normalizing the tristimulus values by their sum, gives a new set of values that are independent the luminance, and thus, only specify the color. Doing so yields a new color-space with coordinates *x*, *y*, and *z* (by convention), which are generally known as chromaticity coordinates. To avoid confusion with spatial coordinates in Figs. 1 and 6, these are specified here as $x_{\text {c}}$, $y_{\text {c}}$, and $z_{\text {c}}$. Being normalized, $x_{\text {c}}+y_{\text {c}}+z_{\text {c}}=1$, only two coordinates are needed to specify any color, $(x_{\text {c}},y_{\text {c}})$, i.e., a single point in a 2D plot, the chromaticity plot, see Ch. 7 in Choudhury^54^. Represented in this way, the color information is presented independent of its luminance. This is an advantage since the colors present in an image can be analyzed regardless of the intensity displayed by any given color.

### Aerosol material and generation

Dry powders of materials are dispersed by the aerosol generator shown in Fig. 6A. The generator consists of a round-bottom flask with an added side port and both ports are threaded. Glass tubes pass-through the ports via sealed holes in screw-on caps. The powder to be aerosolized is placed in the flask and pressurized air is fed to the side-port tube, which has a taper at the end inside the flask such that it acts as a simple nozzle. Air from the nozzle disperses the powder, which then escapes through the top-port tube. To prevent the dispersed particles from affixing to the inner flask surface and to improve their ability to be dispersed, the flask is suspended in an ultrasonic water-bath (Bransonic CPX1800H). Generated aerosol then flows along flexible tubing to a sheath-flow nozzle extending along the *y*-axis and positioned several millimeters above the sensing volume.

The nozzle consists of two nested, co-axial, tapered glass tubes of outer diameter 2 mm and 5 mm, respectively. Aerosol from the generator flows under positive pressure through the inner tube while clean air is supplied to the larger outer tube such that it flows in the annular region surrounding the inner tube; this is the sheath flow^60^. By adjusting the relative pressure of the feed air to the generator and that of the sheath flow, the particles exit the nozzle surrounded by approximately one centimeter of annular laminar air flow, and thus, follow an approximately linear trajectory along the negative *y*-axis through the sensing volume. Note that nozzles of this kind can be much improved over what is used here, e.g., see Pan et al.^60^. In particular, metal is often a superior material to use as static charge can be grounded-out preventing the accumulation of charged particles from clogging the nozzle. Glass is used in this work, however, due to the difficulty of machining metal nozzles of this kind.

A variety of materials are used to calibrate and test the backscatter MWDH method. To establish the WB scale factors, $\alpha _{\text {i}}$, white polymer spheres (Cospheric LLC, WPMS-1.00) are used, which have a distribution of size from $45\,\upmu$m to $53\,\upmu$m in diameter. These particles also serve to verify the size-scale depicted in the reconstructed images. To test the basic ability of the method to resolve primary colors, red, green, and blue (Cospheric LLC, REDPMS-0.98, GPMS-0.98, and BLPMS-1.00) polyethylene microspheres of similar size are tested. The results are shown in Fig. 2B. The mineral dust samples in Fig. 4, with the exception of the road dust, are generated by pulverizing rock samples provided by the Kansas State University (KSU), Department of Geology. A mortar and pestle is used to grind the materials and the resulting powder is dried at low-heat and sieved twice to coarsely size-select particles between approximately 40–210 µm in size, see Fig. 3. The road dust sample is collected from a farm access-road near the KSU campus and is similarly processed. The resulting powders are stored in glass vials in a desiccator box to limit the uptake of moisture, which can impede the ability to aerosolize the particles.

## Data Availability

The datasets generated and/or analysed during the current study are not publicly available due excessive file sizes, but are available from the corresponding author upon reasonable request.

## References

[1] Kulkarni, P., Baron, P. A. & (eds.), K. W. *Aerosol Measurement: Principles, Techniques, and Applications* (Wiley, 2011).

[2] Sorensen CM, Flagan RC, Baltensperger U, Pui DYH (2019). Grand challenges for aerosol science and technology. Aerosol Sci. Tech..

[3] Stocker, T. F. *et al.* IPCC, 2013: Climate change 2013: The physical science basis. in *Contribution of Working Group I to the Fifth Assessment Report of the Intergovernmental Panel on Climate Change* (2013).

[4] Mahowald N (2014). The size distribution of desert dust aerosol and its impact on the earth system. Aeolian Res..

[5] Haywood J, Boucher O (2000). Estimates of the direct and indirect radiative forcing due to tropospheric aerosols: A review. Rev. Geophys..

[6] Karydis VA, Kumar P, Barahona D, Sokolik IN, Nenes A (2011). On the effect of dust particles on global cloud condensation nuclei and cloud droplet number. J. Geophys. Res..

[7] Kumar P, Sokolik IN, Nenes A (2011). Cloud condensation nuclei activity and droplet activation kinetics of wet processed regional dust samples and minerals. Atmos. Chem. Phys..

[8] Després VR (2012). Primary biological aerosol particles in the atmosphere: A review. Tellus B: Chem. Phys. Meteorol..

[9] Lighty JS, Veranth JM, Sarofim AF (2000). Combustion aerosols: Factors governing their size and composition and implications to human health. J. Air Waste Manag. Assoc..

[10] Debecker DP, Bras SL, Boissière C, Chaumonnot A, Sanchez C (2018). Aerosol processing: A wind of innovation in the field of advanced heterogeneous catalysts. Chem. Soc. Rev..

[11] Pósfai M, Li J, Anderson JR, Buseck PR (2003). Aerosol bacteria over the Southern Ocean during ACE-1. Atmos. Res..

[12] Fröhlich-Nowoisky J (2016). Bioaerosols in the earth system: Climate, health, and ecosystem interactions. Atmos. Res..

[13] Fennelly, K. P., Acuna-Villaorduna, C., Jones-Lopez, E., G.Lindsley, W. & Milton, D. K. Microbial aerosols: New diagnostic specimens for pulmonary infections. *Chest* **157**, 540 (2020). 10.1016/j.chest.2019.10.012 PMC7062556 31678308

[14] Thakur AK (2020). Patented therapeutic drug delivery strategies for targeting pulmonary diseases. Exp. Opin. Ther. Patents.

[15] Dolovich MB, Dhand R (2011). Aerosol drug delivery: Developments in device design and clinical use. Lancet.

[16] Sorensen C (2001). Light scattering by fractal aggregates: A review. Aerosol Sci. Technol..

[17] Pettit DR, Peterson TW (1983). Coherent detection of scattered light from submicron aerosols. Aerosol Sci. Tech..

[18] Ferri F (1997). Use of a charge coupled device camera for low-angle elastic light scattering. Rev. Sci. Instrum..

[19] Muñoz O (2012). The Amsterdam-Granada light scattering database. J. Quant. Spectrosc. Radiat. Transfer.

[20] Preston TC, Reid JP (2013). Accurate and efficient determination of the radius, refractive index, and dispersion of weakly absorbing spherical particle using whispering gallery modes. J. Opt. Soc. Am. B.

[21] Sumlin BJ, Heinson WR, Chakrabarty RK (2018). Retrieving the aerosol complex refractive index using PyMieScatt: A Mie computational package with visualization capabilities. J. Quant. Spectrosc. Radiat. Transfer.

[22] Waez MS, Eckels SJ, Sorensen CM (2021). Determination of size and complex index of refraction of single particles with elastic light scattering. Appl. Opt..

[23] Piedra P (2019). Particle-shape classification using light scattering: An exercise in deep learning. J. Quant. Spectrosc. Radiat. Transf..

[24] Piedra P (2020). Where is the machine looking? Locating discriminative light-scattering features by class-activation mapping. J. Quant. Spectrosc. Radiat. Transf..

[25] Bohren CF, Huffman DR (1998). Absorption and Scattering of Light by Small Particles.

[26] Blanche P-A (2020). Optical Holography: Materials, Theory and Applications.

[27] Javidi B (2021). Roadmap on digital holography. Opt. Ex..

[28] Kim M (2010). Principles and techniques of digital holographic microscopy. SPIE Rev..

[29] Kreis T (2006). Handbook of Holographic Interferometry: Optical and Digital Methods.

[30] Van Hout R, Katz J (2004). A method for measuring the density of irregularly shaped biological aerosols such as pollen. J. Aerosol Sci..

[31] Berg MJ, Videen G (2011). Digital holographic imaging of aerosol particles in flight. J. Quant. Spectrosc. Radiat. Transf..

[32] Berg MJ, Holler S (2016). Simultaneous holographic imaging and light-scattering pattern measurement of individual microparticles. Opt. Lett..

[33] Kemppinen O, Heinson Y, Berg M (2017). Quasi-three-dimensional particle imaging with digital holography. Appl. Opt..

[34] Berg MJ, Heinson YW, Kemppinen O, Holler S (2017). Solving the inverse problem for coarse-mode aerosol particle morphology with digital holography. Sci. Rep..

[35] Holler, S., Berg, M. J., Kemppinen, O. & Heinson, Y. W. Two-dimensional scattering and digital holography from isolated aerosol particles. in *Computational Imaging III*. Vol. 10669. 106690B (International Society for Optics and Photonics, 2018).

[36] Kumar SS (2019). Automated droplet size distribution measurements using digital inline holography. J. Aerosol. Sci..

[37] Laning JC, Berg MJ (2019). Orthographic imaging of free-flowing aerosol particles. OSA Continuum.

[38] Gaudfrin F, Santos E, Presley D, Berg MJ (2020). Time-resolved imaging of settling mineral dust aerosols with digital holography. OSA Continuum.

[39] Wu X (2018). In-situ characterization of coal particle combustion via long working distance digital in-line holography. Energy Fuels.

[40] Vössing H-J, Borrmann S, Jaenicke R (1998). In-line holography of cloud volumes applied to the measurement of raindrops and snowflakes. Atmos. Res..

[41] Sauvageat E (2020). Real-time pollen monitoring using digital holography. Atmos. Meas. Tech..

[42] Borrmann S, Jaenicke R (1993). Application of microholography for ground-based in situ measurements in stratus cloud layers: A case study. J. Atmos. Ocean. Tech..

[43] Henneberger J, Fugal JP, Stetzer O, Lohmann U (2013). HOLIMO II: A digital holographic instrument for ground-based in situ observations of microphysical properties of mixed-phase clouds. Atmos. Meas. Tech..

[44] Beals MJ (2015). Holographic measurements of inhomogeneous cloud mixing at the centimeter scale. Science.

[45] Kemppinen O, Laning JC, Mersmann RD, Videen G, Berg MJ (2020). Imaging atmospheric aerosol particles from a UAV with digital holography. Sci. Rep..

[46] Garcia-Sucerquia J (2012). Color lensless digital holographic microscopy with micrometer resolution. Opt. Lett..

[47] Sorensen CM, Heinson YW, Heinson WR, Maughan JB, Chakrabarti A (2017). Q-space analysis of the light scattering phase function of particles with any shape. Atmosphere.

[48] Pan Y-L (2022). Review of elastic light scattering from single aerosol particles and application in bioaerosol detection. J. Quant. Spectrosc. Radiat. Transf..

[49] Fu R (2017). Elastic back-scattering patterns via particle surface roughness and orientation from single trapped airborne aerosol particles. J. Quant. Spectrosc. Radiat. Transf..

[50] Pan Y-L (2017). Measurement of back-scattering patterns from single laser trapped aerosol particles in air. Appl. Opt..

[51] Shimobaba T, Ito T (2018). Computer Holography: Acceleration Algorithms & Hardware.

[52] Berg MJ (2022). Tutorial: Aerosol characterization with digital in-line holography. J. Aerosol Sci..

[53] Giri R, Berg MJ (2022). Backscatter multiple wavelength digital holography for color micro-particle imaging. Appl. Opt..

[54] Choudhury AKR (2014). Principles of Colour and Appearance Measurement: Object Appearance, Colour Perception and Instrumental Measurement.

[55] Süsstrunk, S., Buckley, R. & Swen, S. Standard rgb color spaces. in *Color and Imaging Conference*. Vol. 1999. 127–134 (Society for Imaging Science and Technology, 1999).

[56] Bianco V (2018). Strategies for reducing speckle noise in digital holography. Light Sci. Appl..

[57] Gettens RJ, Fitzhugh EW (1974). Malachite and green Verditer. Stud. Conserv..

[58] Ramanath R, Snyder W, Yoo Y, Drew M (2005). Color image processing pipeline. IEEE Signal Process. Mag..

[59] Guild, J. The colorimetric properties of the spectrum. *Philos. Trans. R. Soc. Lond. Ser. A Contain. Pap. Math. Phys. Character* **230**, 149–187. 10.1098/rsta.1932.0005 (1931).

[60] Pan Y-L, Bowersett J, Hill SC, Pinnick RG, Chang RK (2009). Nozzles for focusing aerosol particles.

